# Excessive Iodine Intake During Lactation Is Not Related to the Incidence of Thyroid Disease: A 3-Year Follow-Up Study

**DOI:** 10.3390/nu17030476

**Published:** 2025-01-29

**Authors:** Seon-Joo Park, Do-Kyung Lee, Hae-Jeung Lee

**Affiliations:** 1 Department of Food and Nutrition, College of BioNano Technology, Gachon University, Seongnam 13120, Republic of Korea; chris0825@hanmail.net; 2 Institute for Aging and Clinical Nutrition Research, Gachon University, Seongnam 13120, Republic of Korea; 3 Department of Health Sciences and Technology, GAIHST, Gachon University, Incheon 21999, Republic of Korea; 4 Gachon Biomedical Convergence Institute, Gachon University Gil Medical Center, Incheon 21565, Republic of Korea

**Keywords:** iodine intake, thyroid dysfunction, thyroid hormones, child growth

## Abstract

**Objectives**: This study aimed to investigate the relationship between excessive postpartum iodine intake and the incidence of thyroid disease in mothers, as well as child growth and development. **Methods**: Of 1054 participants in the 2019 nationwide survey that assessed maternal postpartum iodine intake, 684 mothers participated in a follow-up study. Data on maternal thyroid disease incidence and child growth and development from infant or toddler health checkups were collected. Iodine and nutrient intake were assessed using three-day dietary records, and serum thyroid hormones (triiodothyronine (T3), thyroid-stimulating hormone (TSH), and free thyroxine (free T4)) were measured. Relative risks (RRs) were estimated using Poisson regression analysis. **Results:** Among the 684 participants, 23 (3.4%) were diagnosed with thyroid disease by a physician during the follow-up period. The incidence of maternal thyroid disease was not significantly associated with excessive iodine intake, even after adjusting for confounding factors. Additionally, excessive maternal iodine intake was not related to subclinical hypothyroidism in mothers or child growth and development. **Conclusions**: After a three-year follow-up, no relationship was observed between high postpartum iodine intake and the risk of thyroid disease. Large-scale longitudinal studies are required to evaluate the long-term effects of excessive postpartum iodine intake on maternal health and child growth and development.

## 1. Introduction

The Korean population has a higher consumption of seaweed than Western populations because the sea surrounds them on three sides. Additionally, Korean women have traditionally consumed large amounts of seaweed soup during the postpartum period since the Goryeo Dynasty (918–1392), which is believed to have helped with lactation and uterine contractions [1]. Seaweeds contain significant amounts of iodine, an essential component of triiodothyronine (T3) and thyroxine (T4). These hormones regulate numerous physiological processes, including the basal metabolic rate, protein synthesis, early growth, and neural development [2,3].

Sufficient iodine intake during pregnancy is vital to prevent hypothyroidism, trophoblastic and fetal disorders, and neonatal and maternal hypothyroidism [4]. Conversely, insufficient iodine intake by mothers during pregnancy or lactation increases the risk of hypothyroidism [4] and can adversely affect brain development, leading to growth delays in fetuses and infants [5,6,7]. In contrast, excess iodine intake can cause thyroid dysfunctions, such as hyperthyroidism or autoimmune thyroiditis [8]. A previous review reported that excessive iodine intake through postpartum dietary supplements may lead to thyroid disorders, including thyroiditis and thyroid dysfunction, in both mothers and newborns [9]. The World Health Organization (WHO) recommends a daily intake of 250 μg of iodine during pregnancy and lactation for sufficient supplementation [10].

The association between postpartum iodine intake and thyroid dysfunction has been investigated in several studies; however, the results have been inconsistent. Chung et al. [11] reported that excessive iodine intake through breast milk significantly elevates thyroid-stimulating hormone (TSH) levels in premature infants born before 34 weeks, leading to an increased prevalence of asymptomatic hypothyroidism. Conversely, another study evaluating the association between excessive postpartum seaweed consumption and the incidence of subclinical hypothyroidism (SCH) found no significant association between iodine intake and SCH prevalence in Korean women [12].

Several studies have investigated the effects of perinatal iodine administration on the onset of postpartum thyroiditis in Korea [13,14,15]. However, these studies were limited by small sample sizes and short follow-up periods, making it difficult to draw definitive conclusions about the long-term effects of iodine intake on thyroid function during lactation. Research on the association between iodine intake and thyroid dysfunction during lactation is limited, particularly in iodine-rich countries, such as South Korea and Japan.

Therefore, this study was conducted to assess the relationship between postpartum iodine intake and thyroid disorders in mothers, as well as its potential impact on the growth and development of their children. This analysis utilized follow-up data from the 2019 nationwide representative survey on postpartum iodine intake in Korea.

## 2. Materials and Methods

### 2.1. Participants

**Figure 1:**
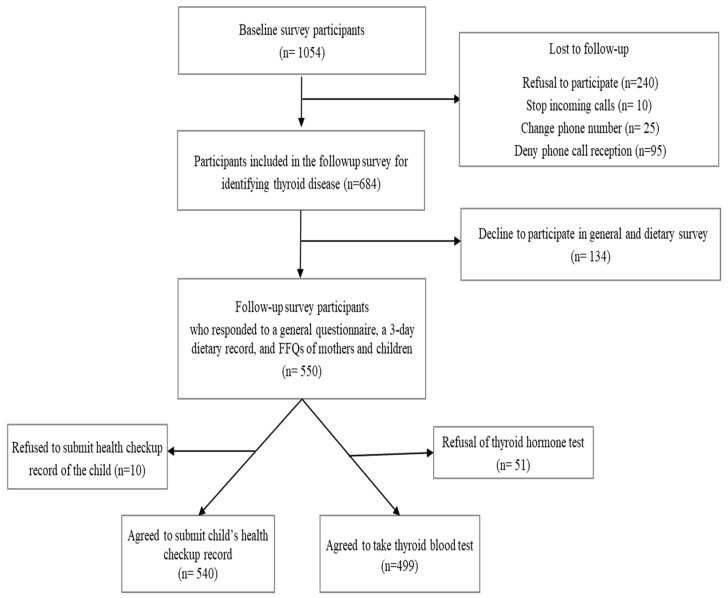
Flowchart of the study participants.

A nationwide representative survey was conducted to investigate postpartum iodine intake levels among Korean women in 2019 [16]. Three years later, we followed up with 1054 Korean women who had initially participated in the study during the first eight weeks postpartum and had no history of thyroid disease diagnosis by a physician. Of the initial 1054 participants, 684 responded to inquiries regarding whether they or their children born in 2019 had been diagnosed with thyroid disease by a physician. Among them, 550 completed detailed three-day dietary records for themselves and their children. Additionally, 499 mothers underwent thyroid hormone tests, and 540 mothers provided infant or toddler health checkup data to assess their children’s growth and development (Figure 1).

This study was approved by the Institutional Review Committee of Gachon University (IRB No. 1044396-202202-HR-042-01, approval date 1 April 2022), and informed consent was obtained from all participants.

### 2.2. Covariates

All data were collected using an online survey system. We used baseline and follow-up data from the mother’s record. The questionnaires for the mothers included age, physical measurements (height and weight), gestational week, children’s height and weight at birth, fertility, feeding type (breastfeeding, mixed feeding, and formula feeding), family history of thyroid diseases, and socioeconomic factors (education level and household income).

### 2.3. Nutrient and Iodine Intake Assessment

Through an online survey, participants submitted dietary records for three nonconsecutive days, including two weekdays and one weekend. These records included details regarding meals and snacks consumed and photos taken before and after meals. The mothers also submitted their children’s three-day dietary records. The researchers monitored the submitted records in real time. They communicated directly with the participants to ensure that all necessary updates were made if any information was missing or if they required further clarification.

The daily nutrient intake of mothers and their children was analyzed using the Computer-Aided Nutrition Analysis Program 5.0 (Can-pro 5.0), developed by the Korean Nutrition Society (Seoul, Korea). Iodine intake was assessed using the iodine database established in the 2019 survey. The methodology for constructing the iodine database was detailed in a previously published paper [16]. To ensure accurate calculation of daily iodine intake, the iodine content of dietary supplements reported by the participants was also included in the analysis.

### 2.4. Thyroid Hormone Measurement and Diagnosis of Thyroid Disease and Subclinical Hypothyroidism

To evaluate thyroid function, serum TSH, free T4, and T3 levels were measured by taking samples from the mothers. We contacted over 50 local hospitals nationwide and directed the participants to the nearest hospital based on their place of residence. Samples were collected by trained nurses, preserved in freezers below −20 °C, and transported within 48 h to the central laboratory (Sure Quest Lab, Yongin, Korea) for analysis. The TSH, free T4, and T3 levels were measured using an electrochemiluminescence immunoassay (Cobas 8000/E801 analyzer; Roche, Mannheim, Germany).

Maternal thyroid disease was defined as physician-diagnosed thyroid disease. Subclinical hypothyroidism was defined as a TSH level of 4.0–10.0 mIU/L with normal free T4 levels (0.93–1.70 ng/dL) and normal T3 levels (80–200 ng/dL) utilizing the normal range of the analyzer [17].

### 2.5. Thyroid Dysfunction and Growth and Development of Children

Children’s thyroid dysfunction was assessed through a survey questionnaire asking whether the child had been diagnosed with congenital thyroid dysfunction by a physician at birth via the “natural metabolic abnormalities screening test”, a routine newborn test in South Korea designed to detect rare metabolic disorders early for timely treatment and whether the child currently had a diagnosis of thyroid disease by a physician.

Data from infant or toddler health checkups of children born in 2019 were collected to investigate the effect of excessive postpartum maternal iodine intake on children’s physical development. The check-up results, including height, height percentiles, weight, weight percentiles, head circumference, and head circumference percentiles, were uploaded into the system by the mothers.

### 2.6. Statistical Analysis

Descriptive statistics for continuous variables representing subject characteristics were presented as means and standard deviations, and categorical variables were shown as n (%). Group differences were analyzed using the chi-square, Fisher’s exact, and Mann–Whitney U tests. Pearson correlation analyses assessed the association between maternal iodine intake and thyroid hormone levels and between postpartum iodine intake and child growth percentiles.

Poisson regression analysis was used to estimate the relative risk (RR) and 95% confidence interval (CI) for the association between maternal postpartum iodine intake and the incidence of thyroid diseases and between average maternal iodine intake and subclinical hypothyroidism. The Poisson regression method was used to calculate the RR owing to the low incidence rate. To evaluate the impact of excessive iodine intake on thyroid disorders in postpartum women, the tolerable upper intake level (UL) of iodine intake for adults in Korea and Japan, which have similar dietary cultures, was used as the criterion for excessive iodine intake. However, as there were only two cases within the 2400–3000 µg/day range, statistical analysis could not be performed with the population divided into three groups. Thus, the risk of thyroid disorders associated with excessive intake was analyzed separately using the two upper intake levels. Age, family history, parity, BMI, and energy intake could potentially be associated with thyroid diseases [18,19,20]. Therefore, the multivariate model was additionally adjusted for age, family history of thyroid disease, fertility, BMI, and energy intake. All statistical analyses were performed using SAS version 9.4 (Statistical Analysis System Institute, Cary, NC, USA), and statistical significance was set at *p* < 0.05.

## 3. Results

During the three-year follow-up period, 23 cases of thyroid disease were identified among the 684 mothers. Two of these were classified as subclinical hypothyroidism, but they were included in the thyroid disease group because they had a doctor-diagnosed thyroid condition.

Table 1 presents the differences in baseline demographic and socioeconomic characteristics of the participants and their children between the normal and thyroid disease groups. The mean age of the normal group was 32.5 ± 3.8 years, while the mean age of the thyroid disease group was 30.5 ± 3.6 years, representing a statistically significant difference (*p* = 0.018). However, no significant differences were observed in the gestational week, child height, or weight at birth in 2019 between the two groups. Additionally, BMI, fertility, feeding type, family history of thyroid disease, educational level, and income level did not differ significantly between the normal and thyroid disease groups.

A comparison of the average daily calorie and iodine intake at birth in 2019 revealed no significant differences between the normal and thyroid disease groups (Table 2). Similarly, a comparison of the average daily calorie and iodine intake in 2022 among the 550 mothers who completed the dietary survey in 2022 showed no significant differences between the two groups. The iodine intake of the normal group was 2960.2 μg at birth and 368.5 μg in 2022, while the thyroid disease group was 2494.1 μg at birth and 524.5 μg in 2022. When comparing the differences in energy and iodine intake between 2019 and 2022 for the normal group and the thyroid disorder group, no significant differences were observed between the two groups.

Table 3 shows the association between postpartum iodine intake and the occurrence of thyroid disease. When the relationship with thyroid disease was analyzed by categorizing participants into two groups (≤UL vs. >UL) using the tolerable upper intake level of iodine in Korea (2400 μg) and Japan (3000 μg), no significant increase in the risk of thyroid disease was observed in the group consuming iodine above the UL.

**Figure 2:**
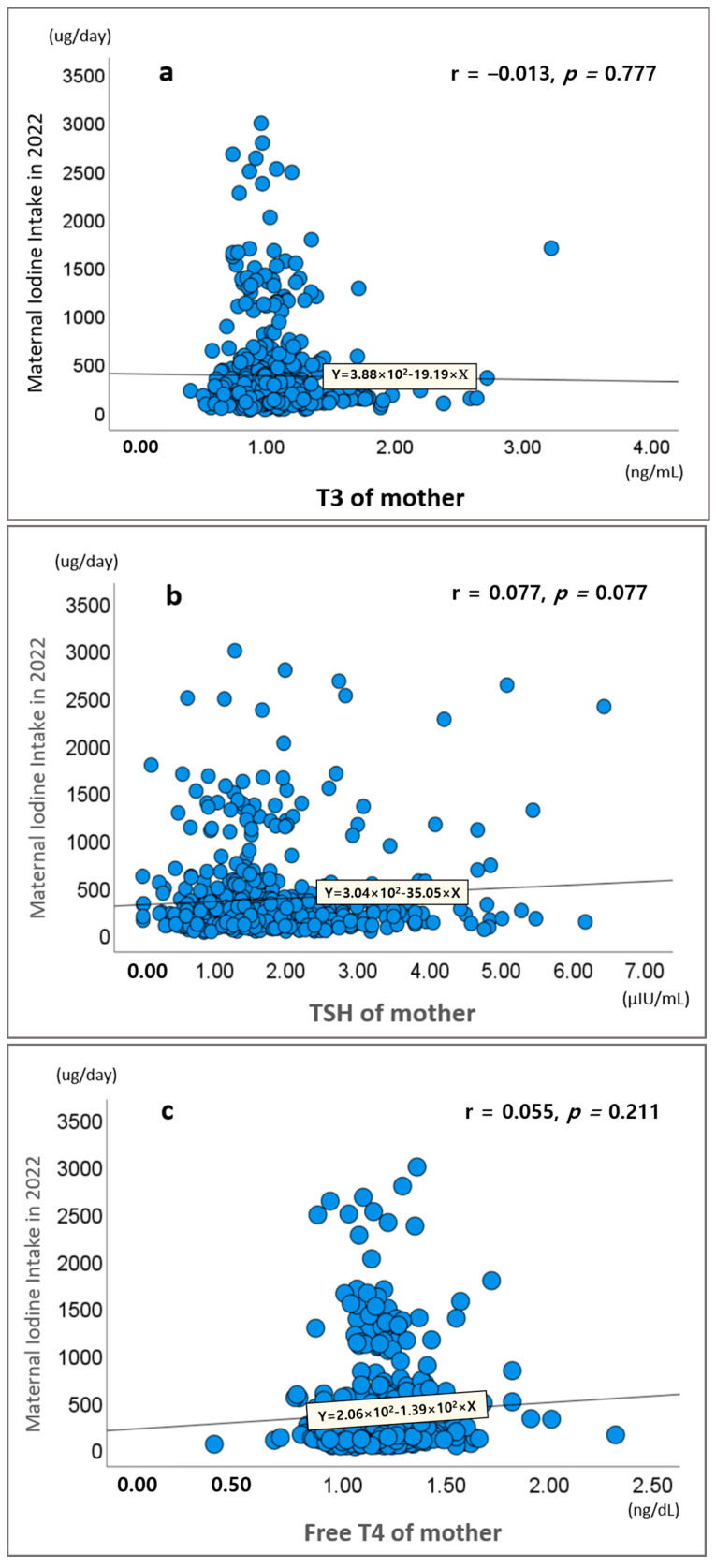
Correlations between maternal iodine intake in 2022 and thyroid hormone levels of mother. (**a**) Correlation between current maternal iodine intake and serum T3. (**b**) Correlation between current maternal iodine intake and serum TSH. (**c**) Correlation between current maternal iodine intake and serum free T4.

Figure 2 shows the correlation between maternal iodine intake and thyroid hormone levels (T3, TSH, and free T4). The analysis revealed no statistically significant correlations between maternal iodine intake and T3 (*r* = −0.013, *p* = 0.777), TSH (*r* = 0.077, *p* = 0.077), or free T4 (*r* = 0.055, *p* = 0.211).

Table 4 shows the results of the analysis of the association between the average iodine intake in 2019 and 2021 and the incidence of subclinical hypothyroidism in mothers. Among the 499 participants who had all three thyroid hormone levels measured, 14 were classified as having subclinical hypothyroidism. After categorizing iodine intake into quartiles and adjusting for all confounding variables, no significant association was observed between iodine intake and the risk of subclinical hypothyroidism in Korean women.

**Figure 3:**
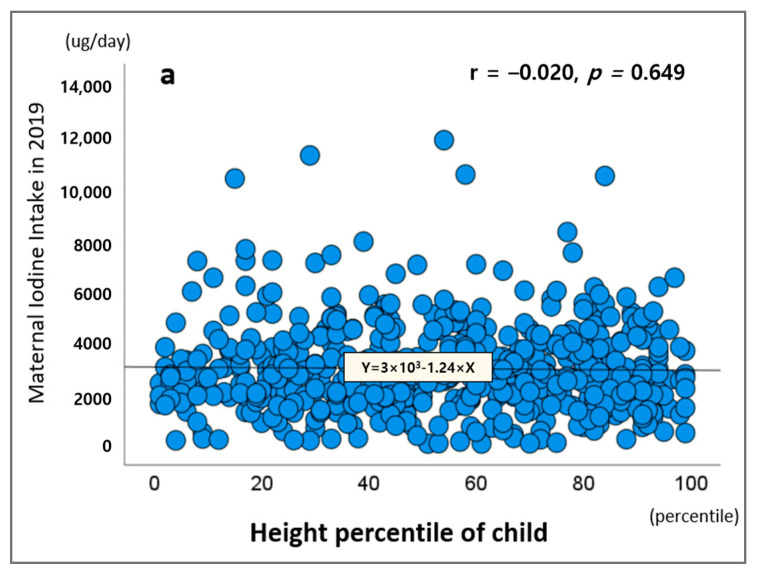

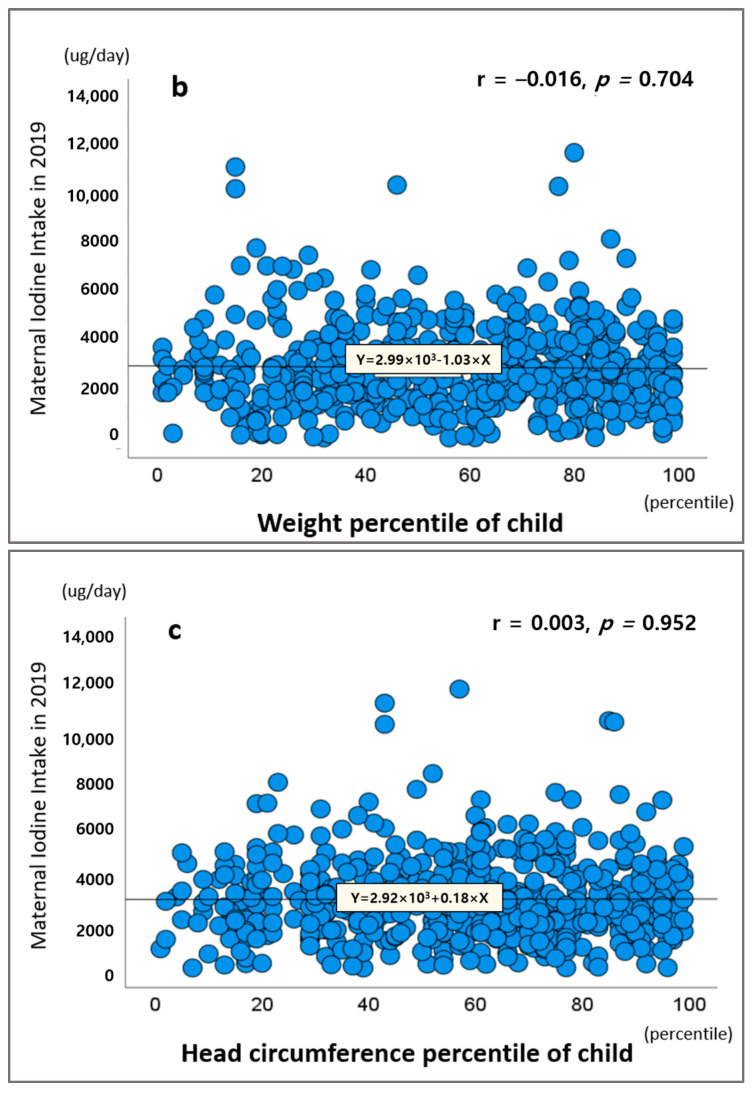
Correlations between postpartum maternal iodine intake and children’s anthropometric percentiles. (**a**) Correlation between postpartum maternal iodine intake and child height percentile. (**b**) Correlation between postpartum maternal iodine intake and child weight percentiles. (**c**) Correlation between postpartum maternal iodine intake and head circumference percentiles.

Figure 3 illustrates the association between postpartum maternal iodine intake in 2019 and child growth status. There were no statistically significant associations observed between maternal postpartum iodine intake and the child’s anthropometric measures, including height percentile (r = −0.020, *p* = 0.649), weight percentile (r = −0.016, *p* = 0.704), and head circumference percentile (r = 0.003, *p* = 0.952).

## 4. Discussion

This study aimed to evaluate the effects of excessive postpartum iodine intake in Korean mothers on maternal thyroid disorders, as well as their children’s growth and development. According to the nationwide representative postpartum iodine intake survey conducted in 2019, the average iodine intake among 1054 postpartum mothers (0–8 weeks) was 2945.6 μg/day [16], which exceeds the WHO recommended intake for nursing mothers (250 μg) by more than tenfold [10]. More than 80% of postpartum iodine intake among Korean mothers is derived from brown seaweed soup (*miyeokguk*). Despite high iodine intake, no significant relationship was observed between postpartum iodine intake and the incidence of thyroid disorders in mothers or the growth and development of their children. A study conducted in Korea reported that iodine intake during pregnancy or postpartum was not associated with maternal thyroid function or the birth weight of their offspring [21], similarly to the finding of our study.

Furthermore, no association was found between the average iodine intake at baseline and the follow-up study and the occurrence of subclinical hypothyroidism in this study. A study examining the association between postpartum seaweed soup consumption and subclinical hypothyroidism in 2046 women from the Korean National Health and Nutrition Examination Survey showed that high iodine intake from seaweed soup during the postpartum period did not significantly increase the risk of subclinical hypothyroidism [12]. A six-month follow-up of 137 Korean mothers shortly after giving birth reported that high iodine intake had no significant influence on the occurrence of postpartum thyroiditis [13]. Another Korean study found no association between high iodine intake and the incidence of postpartum thyroiditis, with no difference in iodine intake between those who developed postpartum thyroiditis and those who did not [15]. In Japan, a study of 514 healthy pregnant women reported a positive correlation between serum TSH levels and urinary iodine concentrations. However, despite the high maternal iodine intake, no adverse effects on fetal maturation were observed within 12 months [22].

Conversely, a three-year follow-up study of 78 Saharan refugee women found a significant association between excessive iodine intake during the postpartum period and the development of thyroid dysfunction, as measured by urinary iodine concentration (UIC) and breast milk iodine concentration (BMIC) [23]. A study involving 34 Japanese infants reported that maternal consumption of large amounts of iodine from seaweed during pregnancy was associated with a diagnosis of hyperthyroidism in children, as determined by their UIC [24]. A study conducted in China involving 7190 women in their first trimester of pregnancy found that UIC within the range of 150 to 249 μg/L was associated with the lowest prevalence of subclinical hypothyroidism and isolated hypothyroidism. UIC levels between 250 and 499 μg/L were associated with a 1.72-fold increased risk of subclinical hypothyroidism, while levels exceeding 500 μg/L were linked to a 2.17-fold increased risk. Notably, UIC levels above 500 μg/L were associated with a 2.85-fold higher risk of isolated hypothyroidism [25]. However, as China’s high iodine intake is primarily through iodized table salt or groundwater with high iodine concentrations, these results may not directly apply to countries like South Korea or Japan, where iodine is primarily consumed through specific foods, such as seaweed.

Although not explicitly focused on mothers, a cross-sectional study in Japan revealed that individuals in regions with high dietary iodine intake exhibited significantly elevated UIC and TSH levels, which correlated with a higher prevalence of subclinical hypothyroidism [26]. Another study that evaluated TSH, thyroid autoantibodies, and urinary iodine levels in 4110 adults from a high-iodine-intake region in Japan suggested that excessive iodine intake may contribute to the onset of latent hypothyroidism [27]. TSH and free T4 levels were significantly higher in the excessive iodine intake group than in the adequate iodine intake group. These findings suggested that excessive iodine intake could potentially contribute to thyroid dysfunction [28].

Excessive iodine intake does not result in severe clinical outcomes [29]. A significant increase in the iodine content of the thyroid gland is regulated by the Wolff–Chaikoff effect, in which thyroid hormone synthesis is temporarily inhibited. Over time, the thyroid adapts, and the sodium–iodide symporter’s (NIS) expression decreases, resulting in a decrease in intrathyroidal iodine concentration and a resumption of thyroid hormone synthesis [30,31]. However, in vulnerable populations, such as individuals with pre-existing thyroid conditions, older adults, fetuses, and newborns, excessive iodine consumption may lead to thyroid dysfunction [32,33].

Nevertheless, previous studies have demonstrated regional differences in the effects of excessive dietary iodine intake on thyroid disorders. Despite adequate iodine intake, Greenland Inuit exhibited a high prevalence of hyperthyroidism and a low occurrence of hypothyroidism, resembling the patterns observed in iodine-deficient populations [34]. This suggests an adaptive mechanism to historically high iodine levels, indicating that prolonged high consumption may lead to metabolic adaptations, allowing the body to manage elevated iodine levels better.

Korean mothers have a unique tradition of consuming seaweed soup after childbirth. Although concerns have been raised regarding the potential risk of thyroid disease due to the iodine content of seaweed soup, it remains uncertain whether Korean mothers increase their risk of thyroid disease by consuming excess iodine from seaweed soup. According to the Korean Dietary Reference Intakes 2020 (KDRI), the upper intake limit for iodine in South Korea is 2400 μg for adults over 19 years. However, there are no standards for pregnant and lactating women [35]. In Japan, the Tolerable Upper Intake Level (UL) of 3 mg/day for pregnant or lactating women has been set at 2 mg/day with an uncertainty factor of 1.5 to guard against excessive iodine intake [36]. Korea also needs to propose a UL for iodine for nursing mothers that considers traditional dietary patterns, such as postpartum consumption of seaweed soup.

In our study, mean iodine intake during the postpartum period was 2960.2 μg/day in the normal group and 2494.1 μg/day in the thyroid disease group. However, three years later, these levels had decreased to 368.5 μg/day and 524.5 μg/day, respectively. Excessive iodine intake during the postpartum period was mainly derived from seaweed soup *(miyeokguk*) consumption, a common dietary practice among Korean mothers. According to another study, among postpartum women consuming seaweed soup daily, the median dietary iodine intakes were 1355 μg/day, 2394 μg/day, and 3063 μg/day when soup was consumed at one, two, and three or more meals, respectively [21]. However, this excessive iodine intake during the postpartum period was not related to maternal thyroid health. Also, over the three-year follow-up period, no infants were diagnosed with thyroid abnormalities, and no association was observed between excessive maternal iodine intake and children’s development and growth.

This study’s strength is that it is the first to investigate the relationship between postpartum maternal iodine intake, maternal thyroid abnormalities, and child growth and development within a nationwide cohort of mothers followed for three years. This study has several limitations. First, the small number of participants with thyroid disease or subclinical hypothyroidism limited the accuracy of risk estimation. Second, only a small proportion of participants (n = 19) consumed iodine below the recommended dietary allowance (RDA) for lactating Korean women (340 µg), which precluded an adequate assessment of the risk of thyroid disease associated with normal iodine intake levels. Third, the follow-up rate was not high, and clinical thyroid function tests were not available in children. Therefore, further studies with larger sample sizes and extended follow-up periods are required to obtain more accurate and comprehensive results.

## 5. Conclusions

This study found that excessive iodine intake during the postpartum period was not associated with maternal thyroid disease, adverse child growth, or developmental outcomes. However, caution is necessary for individuals with pre-existing thyroid abnormalities, as their response to excessive iodine consumption may differ from those with long-term high iodine intake or iodine deficiency.

## Figures and Tables

**Table 1:** Comparison of baseline demographic and socioeconomic characteristics of participants and their children between the normal and thyroid disease groups.

Variables		Normal (*n* = 661)		Thyroid Disease (*n* = 23)		*p*-Value
Age (years)		32.5 ± 3.8 ^(1)^		30.5 ± 3.6		0.018 ^†^
Body mass index (kg/m^2^)		24.1 ± 3.3		24.3 ± 3.2		0.631 ^†^
Child characteristics at birth	Gestational (weeks)	38.6 ± 2.6		38.7 ± 1.5		0.780 ^†^
	Height (cm)	50.1 ± 3.7		50.4 ± 3.1		0.383 ^†^
	Weight (kg)	3.24 ± 0.40		3.34 ± 0.40		0.067 ^†^
Fertility	Primiparity	476	(72.0) ^(2)^	19	(82.6)	0.264 ^‡^
	Multiparity	185	(28.0)	4	(17.4)	
Feeding type	Breastfeeding	137	(20.7)	6	(26.1)	0.656 ^§^
	Mixed feeding	479	(72.5)	15	(65.2)	
	Formula feeding	45	(6.8)	2	(8.7)	
Family history of thyroid disease	Yes	108	(16.3)	6	(26.1)	0.250 ^§^
	No	553	(83.7)	17	(73.9	
Education	Middle School	3	(0.5)	0	(0.0)	0.650 ^§^
	High School	57	(8.6)	1	(4.3)	
	College graduation	526	(79.6)	21	(91.3)	
	Graduate School	75	(11.3)	1	(4.3)	
Household income (USD)	<2000	42	(6.4)	2	(8.7)	0.305 ^§^
	2000–4000	373	(56.4)	10	(43.5)	
	4000–6000	159	(24.1)	9	(39.1)	
	>6000	87	(13.2)	2	(8.7)	

^(1)^ Mean ± standard deviation, ^(2)^ n (%), ^†^ Mann–Whitney U test, ^‡^ chi-square test, ^§^ Fisher’s exact test.

**Table 2:** Comparison of calorie and iodine intake in 2019 and 2022 between the normal and thyroid disease groups.

**Variables**	**Normal ** ** (*n* = 661)**			**Thyroid Disease ** ** (*n* = 23)**			***p*-Value ** **^ †^**
	**Mean**	**±**	**SD**	**Mean**	**±**	**SD**	
Calorie intake in 2019 (kcal)	2077.2 ± 605.3			2073.4 ± 635.9			0.790
Calorie intake in 2019 (kcal/kg)	33.6 ± 11.1			33.6 ± 11.9			0.846
Iodine intake in 2019 (μg)	2960.2 ± 1743.2			2494.1 ± 1340.3			0.210
	**Normal ** ** (*n* = 530)**			**Thyroid Disease ** ** (*n* = 20)**			
Calorie intake in 2022 (kcal)	1231.0 ± 671.0			1413.4 ± 647.5			0.134
Calorie intake in 2022 (kcal/kg)	26.1 ± 6.7			28.1 ± 7.5			0.181
Iodine intake in 2022 (μg)	368.5 ± 461.5			524.5 ± 479.4			0.774
Difference in calorie intake between 2019 and 2022 (kcal)	517.7 ± 640.4			463.5 ± 715.2			0.948
Difference in iodine intake between 2019 and 2022 (μg)	2605.7 ± 1783.0			1938.2 ± 1668.2			0.101

^†^ Mann–Whitney U test; SD: standard deviation.

**Table 3:** Relative risk (RR) and 95% confidence interval (CI) for thyroid disease based on the tolerable upper intake level of iodine in Korea and Japan.

		Postpartum Dietary Iodine Intake (μg)		
	≤2400	>2400	≤3000	>3000
Median iodine intake (μg)	1686	3630	1940	4075
Number of cases/total	13/288	10/396	15/403	8/281
Model 1 RR (95% CI)	1.00	0.43 (0.10–1.82)	1.00	0.81(0.19–3.48)
Model 2 RR (95% CI)	1.00	0.60 (0.13–2.67)	1.00	1.01 (0.23–4.45)
Model 3 RR (95% CI)	1.00	0.92 (0.19–4.51)	1.00	1.66 (0.33–8.22)

Model 1: unadjusted; Model 2: adjusted for age; Model 3: adjusted for age, family history of thyroid disease, fertility, BMI, and energy intake.

**Table 4:** Relative risk (RR) and 95% confidence interval (CI) for subclinical hypothyroidism according to quartile of average dietary iodine intake from baseline and follow-up study.

	Average Dietary Iodine Intake from Baseline and Follow-Up (μg)			
	Q1	Q2	Q3	Q4
Median iodine intake (μg)	756	1265	1758	2631
Number of cases/total	3/124	3/125	2/125	6/125
Model 1 RR (95% CI)	1.00	2.78 (0.46–16.70)	0.98 (0.24–3.93)	2.72 (0.46–15.92)
*p* for trend			0.2646	
Model 2 RR (95% CI)	1.00	2.87 (0.48–17.29)	0.87 (0.21–3.55)	2.29 (0.39–13.46)
*p* for trend			0.2601	
Model 3 RR (95% CI)	1.00	2.45 (0.39–15.31)	0.58 (0.12–2.79)	1.05 (0.19–5.80)
*p* for trend			0.3964	

Model 1: unadjusted; Model 2: adjusted for age; Model 3: adjusted for age, family history of thyroid disease, fertility, BMI, and energy intake.

## Data Availability

Data are available upon request due to restrictions.
